# Study of factors related to the attitudes toward studying abroad among preclinical/clinical undergraduate dental students at three dental schools in Japan

**DOI:** 10.1002/cre2.114

**Published:** 2018-07-27

**Authors:** Hiroko Oka, Yoko Ishida, Guang Hong

**Affiliations:** ^1^ Department of Global Dental Medicine, Graduate School of Biomedical & Health Sciences Hiroshima University Japan; ^2^ Graduate School of Medical and Dental Sciences Niigata University Japan; ^3^ Liaison Center for Innovative Dentistry, Graduate School of Dentistry Tohoku University Japan

**Keywords:** personality, studying abroad, undergraduate dental education, undergraduate dental students

## Abstract

Despite of their general interests in studying abroad, there are two types of dental student in Japan. They are those who decide to go studying abroad and those who do not. The aim of this study was to clarify the factors related to the attitudes toward studying abroad among preclinical/clinical undergraduate dental students who attended three dental schools in Japan. A questionnaire was used to assess undergraduate dental students' attitudes toward studying abroad. We analyzed the differences between junior (preclinical) students and senior (clinical) students on concerns, and relationships between personality traits based on the Big Five and experiences related to studying abroad. Four hundred and thirty‐nine undergraduate students completed the questionnaire. The senior dental students were more worried, more than the junior students, about “lack of language ability in daily life,” “higher quality facilities and environment,” and valued “dental/medical knowledges and skills” and “advantage for getting a job” through studying abroad. Both junior and senior students with experience of/plans for studying abroad reported significantly higher levels of openness to experience than those without such experiences or plans. The less‐open group was more concerned with “lack of language ability in daily life/academic fields,” “higher quality facilities and environment,” “life in a foreign country,” “interpersonal relationships in foreign countries,” and “no friends in foreign countries” than the open‐group. Although the preclinical and clinical dental students at the three dental schools in Japan had different views of concern for studying abroad, “openness to experience” might be a common key factor related to their studying abroad. However, apart from any personality factor, alleviating students' concerns regarding daily life adjustments might be effective in promoting a willingness to go studying abroad.

## 1 INTRODUCTION

In Japan, undergraduate schools including dental schools have conducted various international exchange programs for undergraduates that aimed to help students learn the importance of mutual understanding across multicultural backgrounds in facing rapid globalization (History of International Exchanges, 2014; Japan Students Service Organization, 2016). However, past studies have shown that relatively few Japanese undergraduate students go abroad although they are interested in studying in foreign countries in general (National Federation of University Co‐operative Associations, 2014). For instance, one recent study revealed that although 276 of 418 freshmen from a Japanese undergraduate sample wished to study abroad, only 70 students made attempts to do so (i.e., gather informational materials; Kojima et al., 2014). For undergraduate dental students in particular, in Japan, we reported in a previous study by the survey in three national dental schools that undergraduate dental students, despite expressing an interest in studying abroad and recognizing the value of international exchange opportunities, had several concerns with studying abroad, specifically in relation to language barriers and knowledge of dentistry (Kojima et al., 2014). Moreover, undergraduate dental education in Japan is a 6‐year program; which consists of liberal arts and the preclinical dental program (the period from the first to the fifth grade) and the undergraduate clinical dental program (the period of the fifth and sixth grade after the qualified common computer test and OSCE). There might be differences on the view for studying abroad between preclinical dental students and clinical dental students in Japan.

According to occupational choice theory (Holland, 1984), people in the same occupation group tend to have similar personalities. The Big Five is known as a popular conceptual model of personality and hierarchically. It organizes traits into the broad domains: extraversion, agreeableness, conscientiousness, emotional stability, and openness to experiences (Markon, Krueger, & Watson, 2005; Oshio, Abe, & Cutrone, 2012). To assess the Big Five, there were several well‐stablished instruments such as the Big Five Inventory (Benet‐Martínez & John, 1998; John & Srivastava, 1999), the NEO Five‐Factor Inventory (Costa Jr. & McCrae, 1992), and Ten Item Personality Inventory (TIPI; Gosling, Rentfrow, & Swann Jr., 2003). Measures of the Big Five traits have accumulated compelling empirical support and have been validated within several cultures (Allik & McCrae, 2004; Bornstein et al., 2007; Schmitt, Allik, McCrae, & Benet‐Martínez, 2007). Presently, the Big Five factor structure has provided the basis for comparing, contrasting, and integrating diverse sets of personality traits based on the compelling empirical support and validation within several cultures (Funder, 2001; McCrae & Terracciano, 2005).

In this study, we analyzed results from a questionnaire survey assessing attitudes toward studying abroad and personality among junior (preclinical) and senior (clinical) undergraduate dental students in order to determine potential factors underlying a desire (or resistance) to engaging in a foreign exchange experience.

## 2 METHODS

The study was performed in accordance with the World Medical Association Declaration of Helsinki, and the experimental protocol and consent procedure were approved by the ethical committee of XXX University (Epd‐1097‐1) before starting the study. We used a questionnaire “An Attitude Survey on Studying Abroad” administered to second‐ through sixth‐year students (*n* = 776) from three national university dental schools established in the same year and located in different areas (East, Central, West) within Japan (Oka, Ishida, Hong, & Nguyen, 2018). In the three schools, undergraduate dental students mainly study liberal arts in the first year, then they mainly study in the preclinical and undergraduate clinical program for around 5 years. All of the participants were explained that questionnaire submission shall be deemed to consent for this study. The questionnaires were all self‐administered, unlinkable‐anonymized, and collected immediately after completion. The period of the questionnaire survey was from December 2014 to February 2015, during the later semester in the academic calendar of the each school. At that time, all of the fifth‐year students in the three dental schools had already started their clinical program.

The questionnaire asked about interest in, and prior experience of, or plans for studying abroad. The questionnaire included a section that asked students for their views on the difficulties and benefits of studying abroad with possible answers using a 7‐point scale from 1 (*strongly disagree*) to 7 (*strongly agree*). The scale‐point 4 indicated *neutral*. Participants were divided into junior group (preclinical dental students; second year to fourth year) and senior group (clinical dental students; fifth and sixth year).

The questionnaire contained the Japanese version of the TIPI (TIPI‐J), which was used to assess the Big Five traits (Oshio et al., 2012). The TIPI is a 10‐item questionnaire that consists of two items for each personality trait and non‐English versions were developed like in German (Muck, Hell, & Gosling, 2007), Dutch (Hofmans, Kuppens, & Allik, 2008), and Japanese (Oshio et al., 2012). In the TIPI‐J, responses were made on scales that ranged from 1 (*strongly disagree*) to 7 (*strongly agree*), and the Big Five traits were calculated as the scores 2 to 14 according to the manual (Oshio et al., 2012). The dental students were also divided into two groups based on responses to the openness to experience items from the TIPI‐J: scores higher than 1 *SD* (2.39) from the mean (8.01) were considered “open,” and those with scores lower than 1 *SD* were considered “less‐open.” The answers were then compared between the two groups using Fisher's exact tests. Welch's *t* test was also used to analyze group differences. R (The R Foundation for Statistical Computing, Vienna, Austria) was used for all statistical analyses. A *P* value of <0.05 was considered statistically significant.

## 3 RESULTS

After excluding participants with incomplete data, a total of 439 valid responses were obtained (57.31% of the targeted sample). Sample characteristics are shown in Table 1A.

**Table 1a:** Distribution of dental students according to gender and course year

	Year of course	Male	Female	N/A	Total	(%)
Junior (preclinical)	2	55	53	0	108	64.29
	3	33	31	0	64	43.54
	4	46	47	0	93	65.49
	Total	134	131	0	265	
Senior (Clinical)	5	44	47	2	93	59.62
	6	35	46	0	81	52.94
	Total	79	93	2	174	
	Total	213	224	2	439	57.31

Associations regarding interest in and prior experience/plans for studying abroad, there were no differences between the junior and senior groups (Table 1B). The senior group had significantly higher scores in “lack of language ability in daily life” and “higher quality of facilities and environment” as concerns (Table 2), and valued more about “dental/medical knowledges and skills” and “advantage for getting a job” (Table 3) than the junior students. On the other hand, the junior group had significantly higher mean scores in “lack of specialized knowledge” (Table 2).

**Table 1b:** Interests in studying abroad (*n* = 439)

	Junior			Senior			*P* (“J” vs. “S”)
	Yes	No	N/A	Yes	No	N/A	
Do you want to study abroad?	177	85	3	108	63	3	0.56
Do you have experiences/plans to study abroad?	48	210	7	39	127	8	0.24

*Note*. Two groups' responses to the questions about desires and experiences related to studying abroad.

**Table 2:** Participants average scores with regard to concerns about studying abroad

	Junior		Senior		*t*	*P* value
	Mean	*SD*	Mean	*SD*		
Lack of language ability in daily life	5.60	1.48	5.96	1.21	−2.77	0.01**
Lack of language ability in academic fields	6.09	1.26	6.25	1.06	−1.41	0.16
The cost of overseas trip and living expenses	5.77	1.42	6.18	5.44	−0.14	0.89
Tuition fees	5.39	1.55	5.54	1.34	−1.13	0.26
Difficulties for the accommodation	5.03	1.50	5.13	1.40	−0.71	0.48
Higher quality of facilities and environment	4.53	1.32	4.82	1.28	−2.24	0.03*
Lower quality of facilities and environment	3.48	1.27	3.74	1.36	−2.01	0.04*
Lack of specialized knowledge	5.59	1.31	5.27	1.36	2.38	0.02*
Delay of the graduation	5.00	1.78	4.83	1.76	0.83	0.41
Complicated procedure for the application	5.18	1.50	5.28	1.33	−0.75	0.46
Lack of information to study abroad	5.28	1.25	5.28	1.33	−0.00	1.00
Worries about life in a foreign country	5.37	1.63	5.37	1.51	−0.00	1.00
Worries about the interpersonal relationship in foreign countries	5.31	1.69	5.24	1.55	0.42	0.67
No friends in foreign countries	5.31	1.80	5.35	1.66	−0.27	0.78
No benefits/meaning from studying abroad	2.91	1.73	3.45	1.82	−3.07	0.00**
No clinical training (participants only do observations)	3.97	1.50	4.63	4.85	−1.75	0.08

*Note*. “Score” represents the average score for each group (1 = *strongly disagree*, 7 = *strongly agree*, 4 = *neutral*). Welch's *t* test:

* *P* < 0.05.

** *P* < 0.01.

**Table 3:** Participants average scores with regard to benefits of studying abroad

	Junior		Senior		*t*	*P* value
	Mean	*SD*	Mean	*SD*		
Language skills	6.16	1.03	6.07	1.02	0.86	0.39
Cultural communication skills	6.15	1.01	6.08	1.00	0.71	0.48
International view of thinking	6.07	1.11	5.96	1.10	1.05	0.29
Dental/medical knowledges and skills	5.22	1.36	5.49	1.15	−2.24	0.03*
International networking	5.74	1.12	5.67	1.23	0.58	0.56
Advantage for getting a job	5.11	1.42	5.41	1.27	−2.29	0.02*
Intercultural communication	6.05	0.97	6.02	0.99	0.33	0.74
Self‐development	5.87	1.28	5.89	1.14	−0.18	0.85
Identity as a Japanese person	5.20	1.44	5.33	1.26	−0.96	0.34
Understanding for advanced research	5.29	1.36	5.42	1.15	−1.20	0.27

*Note*. “Score” represents the average score for each group (1 = *strongly disagree*, 7 = *strongly agree*, 4 = *neutral*). Welch's *t* test:

* *P* < 0.05.

**Figure 1:**
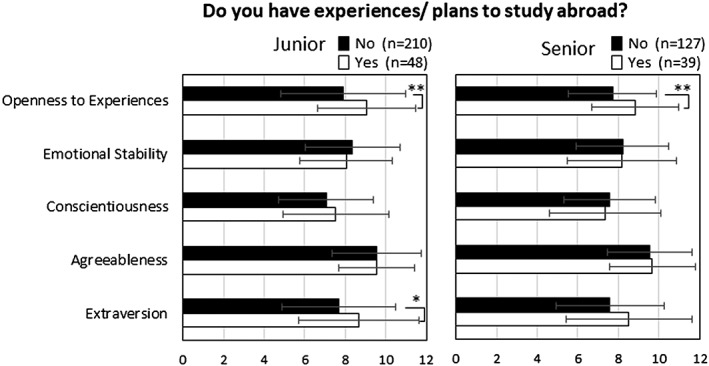
Big Five personality traits among dental students who decided to study abroad. (Junior: *n* = 258, Senior: *n* = 166) Welch's *t* test: *P < 0.05, **P < 0.01

Respondents with experience of or plans to study abroad reported significantly higher openness to experience scores (*P* < 0.01) compared with individuals without experience of or plans to study abroad (Figure 1). When compared with the open group, the less‐open group was more worried about “lack of language ability in daily life,” “lack of language ability in academic fields,” “higher quality of facilities and environment,” “life in a foreign country,” “interpersonal relationships in foreign countries,” and “no friends in foreign countries” (Table 4). Finally, regarding the benefits of studying abroad, there were no significant differences on the ratings between the open and less‐open groups (data not shown).

**Table 4:** Openness to experience and participants average scores with regard to concerns about studying abroad

	Open group		Less‐open group		*t*	*P* value
	Mean	*SD*	Mean	*SD*		
Lack of language ability in daily life	4.89	1.88	6.08	1.33	4.09	0.00***
Lack of language ability in academic fields	5.83	1.42	6.35	1.29	2.15	0.03*
The cost of overseas trip and living expenses	5.58	1.75	5.64	1.61	0.22	0.83
Tuition fees	5.45	1.71	5.21	1.67	−0.80	0.42
Difficulties for the accommodation	4.66	1.76	4.98	1.58	1.07	0.29
Higher quality of facilities and environment	4.19	1.44	4.78	1.38	2.36	0.02*
Lower quality of facilities and environment	3.27	1.22	3.12	1.18	−0.69	0.49
Lack of specialized knowledge	5.42	1.48	5.59	1.56	0.62	0.54
Delay of the graduation	4.91	1.77	4.95	2.09	0.13	0.90
Complicated procedure for the application	4.94	1.69	5.25	1.66	1.04	0.30
Lack of information to study abroad	5.36	1.36	5.42	1.39	0.23	0.82
Worries about life in a foreign country	4.59	1.85	5.75	1.70	3.62	0.00***
Worries about the interpersonal relationship in foreign countries	4.59	1.99	5.78	1.45	3.82	0.00***
No friends in foreign countries	4.72	2.07	5.75	1.75	3.00	0.00***
No benefits/meaning from studying abroad	2.55	1.74	3.48	1.97	2.80	0.01**
No clinical training (participants only do observations)	4.00	1.83	4.15	1.74	0.47	0.64

*Note*. Open group: openness to experience score higher than 1 *SD* from the mean. Less‐Open group: openness to experience score lower than 1 *SD* from the mean. “Score” represents the average score for each group (1 = *strongly disagree*, 7 = *strongly agree*, 4 = *neutral*). Welch's *t* test:

* *P* < 0.05.

** *P* < 0.01.

*** *P* < 0.001.

## 4 DISCUSSION

The purpose of this study was to clarify the factors related to the attitudes toward studying abroad among preclinical/clinical undergraduate dental students. No significant relations between the studying stage of undergraduate students and their interest in/experiences with studying abroad were found. Additionally, this study observed that both the preclinical and clinical students had higher scored concerns regarding language barriers especially in academic fields than other concerns. Nevertheless, there were significant differences on the view related to studying abroad between the preclinical dental students and the clinical dental students: the preclinical students were more worried about their “lack of specialized knowledge,” on the other hand, the clinical students valued more about “dental/medical knowledge and skills” and “advantage for getting a job” as the benefits of studying abroad. There might be two reasons for this. First, although participants were dental students who had already begun to specialize, they were at different stages in their undergraduate program. The students in the senior group had already passed the qualified common computer‐based test about basic dental knowledge and started the clinical program in hospitals, whereas the junior group had not yet done. Second, as these students would be joining activities within medical/dental institutions while studying abroad, students would need to understand field‐specific terms and some may not have acquired this understanding.

This study revealed that the preclinical and clinical dental students who ultimately decided to study abroad both scored higher on measures of openness to experience. Conversely, there were no significant differences regarding levels of agreeableness, emotional stability, and conscientiousness between the students who had decided to go studying abroad and those who had not. Interestingly, although the preclinical dental students who decided to go studying showed higher extraversion scores, there were no significant differences on the scores among the clinical dental students. These findings are in partial accordance with those of a past study assessing first‐year undergraduate students, revealing that students with an inward‐focused personality did not want to study abroad (Kojima et al., 2014). Past studies have suggested that noncognitive factors, such as personality traits, can be developed through extracurricular activities (Cabane & Clark, 2011; Kume, Hanaoka, Mizutani, Ohtake, & Okuyama, 2013; Lleras, 2008). Furthermore, personalities of young adults may also be influenced by social experiences (Roberts, Caspi, & Moffitt, 2003). In a retrospective study of employees, it was reported that participating in group sports during high school was associated with higher extraversion and conscientiousness in adulthood, whereas personal activities promoted “emotional stability and openness to experience” (Kume et al., 2013). Thus, it is possible that experiences during the dental school program, including curricular and extracurricular activities, may actually have impacted on their development of the personality traits or their behavior.

Thus, it may be important to focus on why the more less‐open dental students did not go to study abroad in order to better encourage them to do so. Considering about the concerns of less‐open students in this study, providing experience with English and “international friends” could be one method for helping students develop their confidence, as has been reported in the past (Takehara et al., 2016). Additionally, the less‐open students were concerned with costs and specialized knowledge required to study abroad to the same level as open students. Regarding the benefits of studying abroad, no significant differences between the open/less‐open groups were found and all of the scores were more than 4 *neutral* (data not shown). In a previous study, it was observed that dental undergraduate students with a desire to study abroad were worried about language barriers, costs, and their dental knowledge (Kojima et al., 2014). Overall, it seems that targeting dental students' language concerns within the academic realm is important. Moreover, the provision of information about daily life and costs would be effective to motivate the dental students to go studying abroad.

A few study limitations should be noted. First, although 512 individuals responded to the questionnaire, around 70 did not complete the personality measure. The questionnaire consists of four sections grouped in two sheets (Sections 1–2 and Sections 3–4) and many of the students only fulfilled the first page not the personality measure in the last section of the second page. Thus, the overall valid response rate was under 60% (57.31%). Second, a detailed examination of differences between extraversion and openness to experience groups was not conducted. However, a significant and positive correlation between introversion and openness to experience (data not shown) was found. In the future, it might be useful to conduct a qualitative analysis of students with experience studying abroad in order to obtain more specific profiles of exchange student characteristics.

## 5 CONCLUSION

Although the preclinical and clinical students from the three national dental schools in Japan had different concerns about studying abroad, “openness to experience” might be potential factors related to interest in studying abroad among the undergraduate dental students. However, apart from any personality factor, many of the dental students recognized the benefits of studying abroad, including opportunities to develop language skills, multicultural understanding, and professional knowledge and skills. Alleviating students' concerns regarding daily life adjustments might be effective in promoting a willingness to go studying abroad.

## CONFLICT OF INTERESTS

The authors have no conflicts of interest to declare. This research did not receive any specific grant from funding agencies in the public, commercial, or not‐for‐profit sectors.
